# Poor Colonoscopy Bowel Prep in 69‐Year‐old with Mild Neurocognitive Disorder: A Case Example

**DOI:** 10.1002/alz.088265

**Published:** 2025-01-09

**Authors:** Yonah Joffe, Kara Eversole, Juliana S Burt, Faith Kimmet, David Estores, Jared J. Tanner, Franchesca Arias, Catherine C. Price

**Affiliations:** ^1^ University of Florida, Gainesville, FL USA

## Abstract

**Introduction:**

Colonoscopies are medical procedures used to identify colon abnormalities and remove polyps to decrease the incidence of colorectal cancer. Prior to this exam, patients must undergo bowel preparation to ensure proper cleansing of the colon and maximize outcomes (e.g., full identification of pre‐cancerous polyps). Many risk factors for poor bowel preparation have been identified (e.g., age, low health literacy). However, there has been minimal consideration of preoperative mild or major neurocognitive disorders as a risk factor. A 2019 study by Arias and colleagues found that 68% of older adults undergoing colonoscopies met criteria for neurocognitive disorders. For these patients, inadequate bowel preparation was identified in 100% of those with major neurocognitive disorder and 28% with mild neurocognitive disorder. The present case study provides another example regarding the importance of cognition when considering colonoscopy preparation.

**Case Presentation Demographics:**

CR is a 69‐year‐old non‐Hispanic White male with 13 years of education who worked in the oil field, then as an auto‐mechanic, and later owned an auto repair business. Neuropsychological Testing: Mild neurocognitive disorder, amnestic type, with rapid forgetting and impaired semantic fluency coupled with average attention, working memory, and processing speed. Lives with spouse and is largely independent in functional abilities. Medical records indicate history of postoperative delirium and cardiovascular history. Brain MRI shows white matter abnormalities. Problem: CR was scheduled to complete a routine colonoscopy screening. Review of medical records report colonoscopy was a failed procedure due to poor bowel preparation. Gastroenterology rating of preparation with the Boston Bowel Preparation Scale (BBPS) totaled to 4, indicating inadequate bowel preparation (0‐9, 0 = suboptimal; 9 = optimal). Outcome: CR was referred to undergo a second colonoscopy.

**Discussion:**

This case highlights how preoperative cognitive impairment and the possibility of neurodegenerative disease (i.e., presence of white matter abnormalities) is an important consideration for individuals referred for routine colonoscopy procedures. The case is consistent with the work from Arias et al. (2019) and indicates the need for larger prospective examinations on cognitive risk for elective “routine” procedures involving preparation that involves patient planning. Supported by: K07AG066813.

